# Upregulated IL-1β in dysferlin-deficient muscle attenuates regeneration by blunting the response to pro-inflammatory macrophages

**DOI:** 10.1186/s13395-015-0048-4

**Published:** 2015-08-07

**Authors:** Tatiana V. Cohen, Gina M. Many, Bryan D. Fleming, Viola F. Gnocchi, Svetlana Ghimbovschi, David M. Mosser, Eric P. Hoffman, Terence A. Partridge

**Affiliations:** Center for Genetic Medicine Research, Children’s National Medical Center, 111 Michigan Avenue NW, Washington, DC 20010 USA; Center for Genetic Muscle Disorders, Kennedy Krieger Institute, 707 N. Broadway, Baltimore, MD 21205 USA; Department of Neurology, Johns Hopkins University School of Medicine, Baltimore, MD 21205 USA; Department of Cell Biology and Molecular Genetics, University of Maryland, College Park, MD 20742 USA

**Keywords:** Skeletal muscle, Myoblasts, Macrophages, Cell-cell interactions, Muscular dystrophy, Dysferlin, LGMD2B

## Abstract

**Background:**

Loss-of-function mutations in the dysferlin gene (DYSF) result in a family of muscle disorders known collectively as the dysferlinopathies. Dysferlin-deficient muscle is characterized by inflammatory foci and macrophage infiltration with subsequent decline in muscle function. Whereas macrophages function to remove necrotic tissue in acute injury, their prevalence in chronic myopathy is thought to inhibit resolution of muscle regeneration. Two major classes of macrophages, classical (M1) and alternative (M2a), play distinct roles during the acute injury process. However, their individual roles in chronic myopathy remain unclear and were explored in this study.

**Methods:**

To test the roles of the two macrophage phenotypes on regeneration in dysferlin-deficient muscle, we developed an *in vitro* co-culture model of macrophages and muscle cells. We assayed the co-cultures using ELISA and cytokine arrays to identify secreted factors and performed transcriptome analysis of molecular networks induced in the myoblasts.

**Results:**

Dysferlin-deficient muscle contained an excess of M1 macrophage markers, compared with WT, and regenerated poorly in response to toxin injury. Co-culturing macrophages with muscle cells showed that M1 macrophages inhibit muscle regeneration whereas M2a macrophages promote it, especially in dysferlin-deficient muscle cells. Examination of soluble factors released in the co-cultures and transcriptome analysis implicated two soluble factors in mediating the effects: IL-1β and IL-4, which during acute injury are secreted from M1 and M2a macrophages, respectively. To test the roles of these two factors in dysferlin-deficient muscle, myoblasts were treated with IL-4, which improved muscle differentiation, or IL-1β, which inhibited it. Importantly, blockade of IL-1β signaling significantly improved differentiation of dysferlin-deficient cells.

**Conclusions:**

We propose that the inhibitory effects of M1 macrophages on myogenesis are mediated by IL-1β signals and suppression of the M1-mediated immune response may improve muscle regeneration in dysferlin deficiency. Our studies identify a potential therapeutic approach to promote muscle regeneration in dystrophic muscle.

## Background

A spectrum of distinct myopathies is associated with mutations in the protein dysferlin [1]. The most common manifestations include limb-girdle muscular dystrophy 2B (LGMD2B), an autosomal recessive myopathy marked by proximal muscle weakness, with an onset in the late teens ([2, 3], reviewed in [4]) and Myoshi myopathy, characterized by a progressive muscle wasting involving distal muscles ([5] reviewed in [4]). In general, these myopathies feature chronic regeneration and fibrosis [6], a selective loss of type 2 muscle fibers and a moderate degree of inflammation surrounding the necrotic fibers [7], although the severity of pathology is variable. Additionally, tissue pathology is focal and sporadic, making dysferlinopathy a challenging disorder to characterize, diagnose, and treat.

Dysferlin-deficient muscle is characterized by inflammatory foci that consist of necrotic fibers and infiltrating immune cells. Myofibers undergoing necrosis release danger-associated molecular patterns (DAMPs) that activate innate immune receptors resulting in release of cytokines and chemokines and subsequent recruitment of inflammatory cells that remove the necrotic debris and facilitate muscle regeneration [8]. The primary infiltrating cells in dystrophic muscle are macrophages, which have been studied more extensively in the *mdx* mouse model of Duchenne muscular dystrophy [9].

Macrophages have been classified into two major categories: the classically activated (M1) and the alternatively activated (M2a). M1 macrophages, identified by expression and secretion of TNFα, Cox-2, IL-1β, IL-12, and iNOS, respond to TLR ligands such as lipopolysaccharide (LPS) and demonstrate phagocytic and bacteriocidal activity [10]. In contrast, M2a macrophages, identified by expression of mannose receptor (Mrc1), resistin-like α (Retnla, Fizz1), and chitinase 3-like 3 (Chi3l3, Ym1), are activated by IL-4 or IL-13 signals that arise during Th2 immune responses and participate in wound-healing processes (reviewed in [11, 10]). Immune cells are sequentially recruited to sites of acute injury, with a wave of neutrophils followed by M1-polarized macrophages that phagocytose necrotic material. M2a macrophages are also recruited at the time of M1 macrophage infiltration [12] and remain at the site of injury as M1 macrophages transdifferentiate to M2a macrophages in situ [13, 14], thereby resolving the injury and promoting myoblast differentiation. Studies of the macrophage populations in *mdx* mice determined that M1 macrophages predominate during early phases to be replaced by M2a macrophages during the later regenerative/fibrotic stages of the disease [15]. Additionally, regulatory macrophages, M2b, are a third category of macrophages and are characterized by IL-10 secretion and anti-inflammatory activity.

The effects of the two types of differentially polarized macrophages on muscle cells were previously studied in the context of wild-type muscle [16]. Direct co-culture of myoblasts with M2 macrophages, or conditioned medium from them, increased myogenin-positive cells and myotubes, whereas co-culture or conditioned medium from M1 macrophages had no effect [16], whereas other studies have suggested that M1 macrophages promote myoblast proliferation and M2a promote myotube fusion [14]. These studies implicate macrophages as playing a major role in the process of muscle regeneration. However, the role of macrophages in chronic myopathies remains to be elucidated.

To examine myoblast-macrophage interactions, we used an *in vitro* co-culture system of macrophages and immortalized myoblasts (H-2K cells), focusing on the molecular effects of macrophage-released soluble factors on myoblasts. We hypothesized that macrophage-secreted factors can influence differentiation of dysferlin-deficient muscle and proceeded to identify such factors and examine their effects on myoblasts. To approach these questions, we (1) examined the effects of co-culture with M1 and M2a macrophages on the differentiation of wild-type (WT) mouse myoblasts, (2) compared the effects of macrophages in dysferlin-deficient dystrophic myoblasts, and (3) identified IL-1β as the M1-derived factor inhibiting muscle differentiation in dysferlin deficiency. Importantly, we show for the first time that use of a blocking antibody to inhibit IL-1β improves muscle differentiation in dysferlin-deficient myotubes. Our studies delineate the effects of the pro-inflammatory environment on muscle regeneration in dysferlinopathy and raise the possibility of modulating this environment to promote muscle regeneration.

## Methods

### Animals

All animal protocols were reviewed and approved by the local Institutional Animal Care and Use Committee of the Children’s National Medical Center, Washington, DC. Four- to seven-month-old Bla/J mice (B6.A-Dysf^prmd^/GeneJ) (stock # 012767) and BALB/c (stock# 000651) were obtained from The Jackson Laboratory (Bar Harbor, ME). The Bla/J model of dysferlin deficiency, produced by crossing the A/J naturally occurring dysferlin-mutated mice onto the C57Bl6/J background, was previously characterized as having moderate inflammation and pathology, making it a suitable murine model for human disease and one that has good strain-specific controls [17]. Mice were euthanized by CO_2_ asphyxiation followed by cervical dislocation, and muscles were flash frozen in melting isopentane (Fisher) cooled in liquid nitrogen.

### Notexin injury

Notexin is a myotoxic phospholipase A2 derived the venom of the Australian tiger snake (*Notechis scutatus*). Four- to seven-month-old Bla/J and C57Bl/6J male mice were anesthetized by isoflurane inhalation, and the hindlimbs were shaved. A unilateral injection of 20 μl of notexin (10 μg/ml; Latoxan, Valence, France) was delivered into the tibialis anterior (TA) muscle, with the contralateral leg used as an uninjected control. To identify injured muscles, tattoo ink at a concentration of 1:6 *v*/*v* was included in the notexin solution. To mark proliferating cells, 5′bromo-2′deoxyuridine (BrdU, Sigma, St. Louis, MO) at 0.8 mg/ml was administered in the drinking water for 1 week from the time of injury and the tissues were harvested at indicated time points.

### Bone marrow-derived macrophage culture

**Fig. 1:**
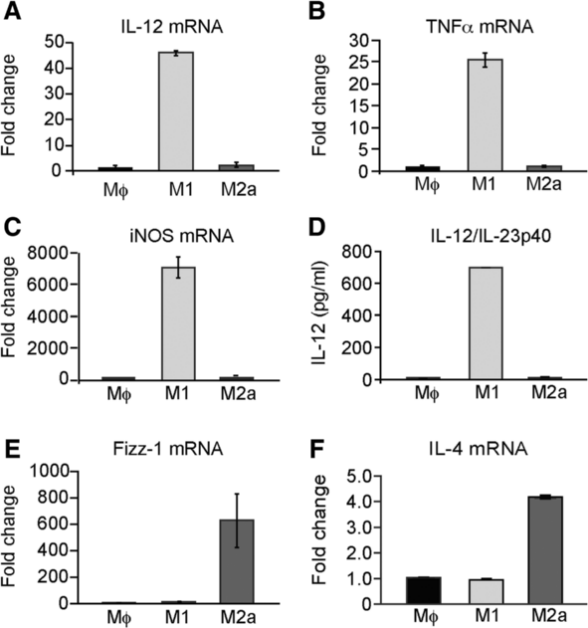
Characterization of M1 and M2 macrophages in response to differential stimuli. Bone marrow-derived macrophages were either unstimulated (Mϕ), stimulated with LPS, to induce M1, or IL-4 to induce the M2a phenotypes, respectively. **a** M1 phenotype validation using qRT-PCR for IL-12, **b** TNFα, and **c** iNOS. **d** ELISA analysis for IL-12/23p40 in M1, M2a, or control (Mϕ) macrophages. **e** Gene expression of *Fizz-1* by qRT-PCR in M1, M2a, or control (Mϕ) macrophages. **f** Gene expression of *IL-4* by qRT-PCR in M1, M2a, or control (Mϕ) macrophages

Bone marrow-derived macrophages were derived as described previously [18] from 6-week-old female BALB/c mice in all cases except experiments using dysferlin-deficient macrophages, which were derived from Bla/J and C57Bl/6 (WT) mice. Briefly, mice were euthanized, and the bone marrow was flushed for collection from the central canals of the femur and tibia with phosphate-buffered saline (PBS) containing 200 U/ml penicillin and 200 μM/ml streptomycin. The cells were plated for culture in Dulbecco’s modified Eagle medium/Ham’s F-12 Nutrient Mixture (DMEM/F-12, Life Technologies, Grand Island, NY) containing 10 % fetal bovine serum (FBS), 100 U/ml penicillin and 100 μM/ml streptomycin, and 1 % L-glutamine (all from Life Technologies) (bone marrow media), supplemented with 15 % L929 cell conditioned media (LCCM) in a 37 °C and 5 % CO_2_-humidified incubator. After 7 days of culture, macrophages were stimulated as previously described to induce classical (M1), alternative (M2a), or regulatory (M2b) macrophage phenotypes [19]. Non-stimulated macrophages were used as controls (Mϕ). The stimulation conditions were as follows: (1) M1 activation, 10 ng/ml LPS (Sigma) in LCCM; (2) M2a activation, 20 ng/ml IL-4 (R&D Systems, Minneapolis, MN) in LCCM; (3) M2b activation, 10 ng/ml LPS plus IgG Ova-immune complex [20] in LCCM; and (4) non-activated, LCCM only. The stimulation protocols were terminated by centrifugation and careful rinsing with PBS to remove all stimulation media. The differentially polarized macrophages were re-suspended in DMEM containing 5 % horse serum, 2 % chick embryo extract (US Biological, Salem, MA), 100 U/ml penicillin, and 100 μM/ml streptomycin (differentiation media). The activated macrophages were validated by quantitative real-time polymerase chain reaction (qRT-PCR) and/or enzyme-linked immunosorbent assay (ELISA) for M1 markers IL-12, TNFα, and iNOS and M2a markers Fizz-1 and IL-4 [18] (Fig. 1).

### H-2K myoblast-macrophage co-culture

Immortalized H-2K myoblasts derived from crosses of Immortomice® with WT and A/J mice were previously described [21]. Myoblasts were maintained in DMEM containing 20 % heat-inactivated FBS, 2 % chick embryo extract, 100 U/ml penicillin and 100 μM/ml streptomycin, and 0.2 % IFNγ (R&D Systems) (proliferation medium). For co-culture differentiation experiments, myoblasts were plated onto Matrigel^™^ (BD Biosciences, San Jose, CA)-coated 6-well dishes at a density of 5 × 10^4^/cm^2^ and incubated at 33 °C and 10 % CO_2_. The next day (day 1 of co-culture), media was changed to differentiation media, lacking IFNγ. In parallel, the differentially polarized macrophages re-suspended in differentiation media were plated onto 0.4-μm Transwell^™^ inserts (Corning #3412, Tewksbury, MA) at 1.5 × 10^6^ cells/insert on top of the myoblasts and the co-cultures were maintained at 37 °C and 5 % CO2. Supernatants were collected, and myotubes were harvested at indicated days of differentiation. All experiments were performed in triplicate.

For cytokine treatments and IL-1β blocking experiments, myoblasts were plated on glass cover slips in 6-well dishes at 0.2 × 10^5^ cells/cm^2^. After an overnight incubation, medium was changed to differentiation medium containing indicated concentrations of IL-4 (PeproTech, Rocky Hill, NJ), IL-1β (R&D Systems), or IL-1β mAb (MM425B, Thermo, Rockford, IL) (day 1). To control for specificity of the blocking antibody, cultures were treated with equimolar concentrations of mouse IgG (Vector Laboratories, Burlingame, CA). Cultures were incubated for three additional days, and then differentiated muscle cells were fixed and immunostained as described below.

### Gene arrays and qRT-PCR

Illumina^®^ Gene arrays were performed on differentiated myoblasts either cultured without macrophages (untreated) or co-cultured with M1 or M2a macrophages for 3 days. All experiments were performed in triplicate. Total RNA was extracted using the Trizol^™^ reagent (Invitrogen, Carlsbad, CA) according to manufacturer’s instructions. Concentration of each RNA sample was determined using a NanoDrop® ND-1000 spectrophotometer (NanoDrop Technologies, Wilmington, DE). The quality of RNA samples was assessed using the Agilent 2100 Bioanalyzer (Agilent Technologies Inc., Santa Clara, CA).

A 200-ng aliquot of high-quality total RNA from each sample was applied for mRNA expression profiling using Illumina® Gene Expression BeadChip Array technology (Illumina, Inc., San Diego, CA). Reverse transcription of the first and synthesis of the second cDNA strands, followed by a single *in vitro* transcription (IVT) amplification that incorporates biotin-labeled nucleotides, were performed using the Illumina® TotalPrep™-96 RNA Amplification Kit (Ambion, Austin, TX). Of the biotin-labeled IVT product (cRNA), 1.5-μg was hybridized to MouseWG-6v2_BeadChip (Illumina, Inc., San Diego, CA) for 16 h, followed by washing, blocking, and streptavidin-Cy3 staining according to the Whole-Genome Gene Expression Direct Hybridization protocol (Illumina, Inc., San Diego, CA). The arrays were scanned using HiScanSQ System, and decoded images were analyzed by GenomeStudio™ Gene Expression Module—an integrated platform for data visualization and analysis (Illumina, Inc., San Diego, CA). GenomeStudio-generated final report table was used in Hierarchical Clustering Explorer software (HCE v3) for filtering, power analysis, and chip-based unsupervised clustering [22].

Significant molecular networks and transcriptional regulators were identified using Ingenuity Pathway Analysis (IPA) software (Ingenuity Systems, Inc., Redwood City, CA). IPA generates networks in which differentially regulated genes can be related according to previously known associations between genes or proteins—the higher the score, the more supportive data is found in the previously published literature (IPA database). Additionally, we used IPA to calculate the z-score to determine expression direction of the transcriptional networks. A z-score >1.5 or <1.5 indicated upregulated or downregulated transcriptional networks, respectively.

qRT-PCR for indicated genes (Table 1) was performed using SYBR® Green PCR Master Mix (Applied Biosystems, Grand Island, NY) on an Applied Biosystems 7900HT Fast Real-Time PCR System. Relative gene expression was determined from absolute Ct values using the ΔΔCt method by normalizing to housekeeping genes, S18 and GAPDH.

**Table 1:** qRT-PCR primers used in the study

	Forward	Reverse
Arg-1	5′-ATGGAAGAGACCTTCAGCTAC-3′	5′-GCTGTCTTCCCAAGAGTTGGG-3′
CCR2	5′-ACACCCTGTTTCGCTGTAGG-3′	5′-CCTGGAAGGTGGTCAAGAAG-3′
Fizz-1	5′-TCCCAGTGAATACTGATGAGA-3′	5′-CCACTCTGGATCTCCCAAGA-3′
Ifng	5′-CATTGAAAGCCTAGAAAGTCTG-3′	5′-CTCATGGAATGCATCCTTTTTCG-3′
IL-1β	5′-TGGGCCTCAAAGGAAAGAAT-3′	5′-CAGGCTTGTGCTCTGCTTGT-3′
IL-4	5′-CATCGGCATTTTGAACGAGGTCA-3′	5′-CTTATCGATGAATCCAGGCATCG-3′
IL-10	5′-CCAGTTTTACCTGGTAGAAGTGATG-3′	5′-TGTCTAGGTCCTGGAGTCCAGCAGAC-3′
IL-12	5′-ATGGCCATGTGGGAGCTGGAG-3′	5′-TTTGGTGCTTCACACTTCAGG-3′
Inos	5′-TGGGAATGGAGACTGTCCCAG-3′	5′-GGGATCTGAATGTGATGTTTG-3′
MCP-1	5′-AGGTCCCTGTCATGCTTCTG-3′	5′-GCTGCTGGTGATCCTCTTGT-3′
Myf5	5′-GCTCGGATGGCTCTGTAGAC-3′	5′-GAACAGCAGCTTTGACAGCA-3′
MyoD	5′-GGCTACGACACCGCCTACTA-3′	5′-GCTCCACTATGCTGGACAGG-3′
Myogenin	5′-CTGACCCTACAGACGCCCAC-3′	5′-TGTCCACGATGGACGTAAGG-3′
TNFa	5′-GTTCTATGGCCCAGACCCTCACA-3′	5′-TCCCAGGTATATGGGCTCATACC-3′
YM-1	5′-GGGCATACCTTTATCCTGAG-3′	5′-CCACTGAAGTCATCCATGTC-3′
S18	5′-TAGCCTTCGCCATCACTGCC TTA-3′	5′-AACCTGGCTGTACTTCCCATCCTT-3′

### Histology and immunofluorescence microscopy

Frozen 10-μm muscle sections were stained with hematoxylin and eosin (H&E) as previously described [23]. For immunofluorescence labeling, sections were fixed with 4 % paraformaldehyde (PFA) and non-specific antibody binding was blocked with a solution containing 20 % normal goat serum, 2 % bovine serum albumen, 0.5 % Triton-X-100, and 0.1 % Tween-20. Sections were incubated with a pan-specific macrophage antibody, F4/80 (AbD Serotec, Raleigh, NC), followed by Alexa 488-conjugated anti-rat secondary antibody. BrdU immunostaining was performed with anti-BrdU (Invitrogen) and anti-laminin (Sigma) antibodies and propidium iodide (PI) to visualize nuclei, as previously described [23]. Quantitation of BrdU-positive nuclei was performed in notexin-lesioned muscles (*n* = 3 mice per time point). Injured muscle sections were visually examined, and 20× images of the lesion epicenter were acquired. Percent BrdU-positive nuclei were calculated by expressing BrdU-positive nuclei as a percentage of total PI-positive nuclei.

Differentiated muscle cells from co-cultures or treatments with indicated cytokines were fixed with 4 % PFA, permeabilized in 0.5 % Triton-X 100/PBS, blocked in 5 % horse serum, and incubated overnight with anti-MyoD (Novus, Littleton, CO) and anti-MyHC (MF20, Developmental Studies Hybridoma Bank, Iowa City, IA) antibodies. The next day, cultures were washed in 0.1 % Triton-X 100/PBS and stained with AlexaFluor® 488-tagged goat anti-rabbit and AlexaFluor® 568-tagged goat anti-mouse secondary antibodies for 1 h at room temperature. Samples were mounted in 4′,6-diamidino-2-phenylindole (DAPI)-containing mounting medium (Vector Laboratories). Desmin immunostaining was performed with the ABC Elite kit (Vector Laboratories) and counterstained with eosin. Myotube fusion was expressed as the number of nuclei in myotubes as a percent of total nuclei in the culture.

Immunofluorescence and histology micrographs were captured at ambient temperature using a Zeiss M2 AxioImager upright epifluorescence microscope using 10×/0.45 N.A. and 20×/0.8 N.A. objectives and an Axiocam Mrm CCD camera. Images were acquired using Axiovision 4.8.2 software and analyzed using ImageJ software (National Institute of Health, Bethesda, MD) [24].

### Cytokine analysis

ELISAs were performed on culture supernatants using the Mouse IL-12/IL-23 p40 Non-allele-specific and Mouse IL-10 Quantikine ELISA kits (R&D Systems) according to manufacturer’s instructions. Cytokine arrays were performed on 1 ml of supernatant from the co-cultures using the Mouse Cytokine Antibody array, Panel A (R&D Systems), according to manufacturer’s instructions. IL-1β and IL-4 analysis of 24-h co-culture supernatants was performed using the MSD Mouse V-PLEX Proinflammatory Panel 1 kit (Meso Scale Diagnostics, Rockville, MD). Briefly, biological triplicates of cell-free supernatants were run in duplicate and analyzed on a QuickPlex SQ120 analyzer using MSD Discovery Workbench 4.0 software (Meso Scale Diagnostics, Rockville, MD). Assay sensitivity using this platform is 0.04 pg/ml.

Whole cell lysates from differentiated muscle cultures were prepared using the NP40 Lysis buffer (Invitrogen) containing anti-proteases (Mini-Complete, Roche, Indianapolis, IN). Protein concentration was determined by the bicinchoninic acid assay (BCA) (Thermo Fisher, Waltham, MA). To assay for IL-1β, 240-ng protein per well was assayed in biological triplicates using the Mouse IL-1β Quantikine ELISA kit (R&D Systems).

### Western blotting

Protein concentration was determined by the BCA assay and 10-μg of protein was resolved on a 4–12 % Bis-Tris SDS-PAGE gel (Life Technologies) and transferred to PVDF membrane (Bio-Rad). Immunoblotting was performed using anti-phosphorylated NFκB p65 subunit (Cell Signaling Technology, Danvers, MA) and anti-vinculin (Sigma) antibodies diluted in 5 % Blocking Reagent (Bio-Rad) in Tris-buffered saline and 0.1% Tween 20 (TBS-T). After washing with TBS-T, membranes were probed with horseradish peroxidase-conjugated goat anti-rabbit secondary antibody (Amersham Biosciences, Piscataway, NJ). The membranes were then incubated with ECL Western Blotting Detection reagent (Pierce, Rockford, IL) and processed on Kodak BioMax XAR X-ray film (Fisher). Densitometry was performed using ImageJ.

### Statistical analysis

Statistical analyses were performed using SigmaPlot v.11 (SyStat Software, Chicago, IL). Student’s *t* test was used to evaluate statistical significance in all analyses where two groups were compared. Unless otherwise specified, two-way analysis of variance (ANOVA) with Tukey post hoc analysis was used to compare more than two experimental groups. Data are presented as means ± standard error of the mean (SEM). *P* value of <0.05 was considered significant.

For gene array analyses, GenomeStudio mRNA expression values were automatically uploaded (*plug-in*) into Partek software (Partek Inc., St. Louis, MO) for statistical analyses and data visualization. During this transaction, Partek automatically applies the robust multi-array average (RMA)—a normalization algorithm—and performs a *log*_*2*_ transformation for the generated expression values. One-way ANOVA was applied to verify significance of the comparative results. Only expression values with fold change ≥1.5 and a *P* value cut-off of ≤0.01 were considered for the further analyses.

## Results

### Chronic macrophage infiltration in dysferlin-deficient muscle attenuates regeneration

**Fig. 2:**
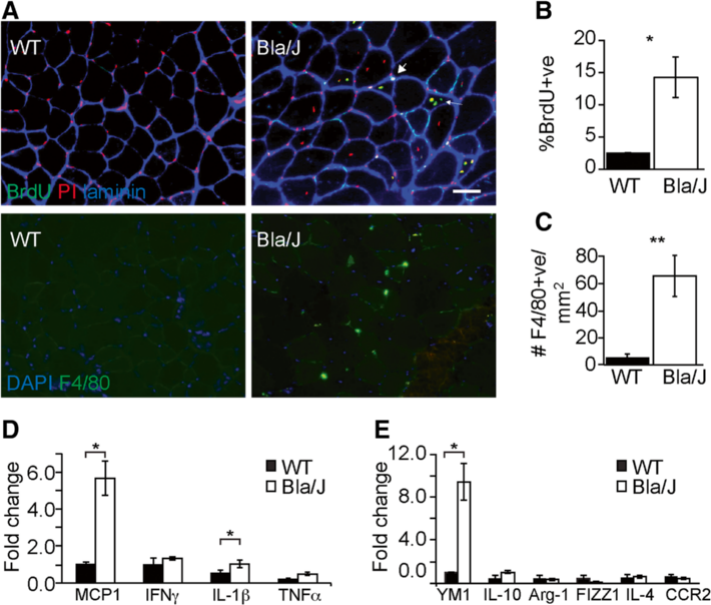
Ongoing satellite cell proliferation and macrophage infiltrate in uninjured Bla/J muscle. **a** Immunofluorescence staining of uninjured 3-month-old WT and Bla/J skeletal muscle. *Upper panels* mice were treated with BrdU in the drinking water for 7 days. Frozen muscle sections were immunostained with anti-laminin (*blue*) and anti-BrdU (*green*) antibodies. Nuclei were detected by propidium iodide (PI, *red*). *Bottom panels* muscle sections were immunostained with an anti-F4/80 (green) antibody and nuclei were detected by DAPI staining (blue). **b** Proliferating cells quantitated in WT and Bla/J muscle are shown as percent of myonuclei that are BrdU-positive (proliferating). *n* = 3 non-overlapping fields from three mice. **c** Quantitation of the number of F4/80-positive macrophages in muscle per mm^2^. *n* = 3 non-overlapping fields each from three mice. **d** Expression of M1-specific markers using qRT-PCR in WT (*black*) and Bla/J (*white*) uninjured muscle. **e** Expression of M2-specific markers in WT (*black*) and Bla/J (*white*) muscle using qRT-PCR. *n* = 3 mice per data point. Data are shown as means ± SEM. *P < 0.05; **P < 0.01. Scale, 50 μm

Dysferlin-deficient muscle contains inflammatory foci consisting primarily of mononuclear infiltrate [25]. To establish a baseline for our studies, we initially validated the published extent of inflammation and ongoing regeneration in adult (4–7-month-old) Bla/J and C57BL6/J (WT) mice using in vivo incorporation of the thymidine analog, BrdU, over a 1-week period to assess the overall cellular proliferation. At this age, Bla/J muscle contained significantly more proliferating cells than WT muscle, (14.0 ± 3.17 vs. 3.0 ± 0.2% *P* < 0.05) (Fig. 2a, b). Of these, 50 ± 14.0 % of the total BrdU-positive cells in Bla/J muscle were central myonuclei, the rest being interstitial, indicating that both muscle and inflammatory cells were proliferating. In contrast, the few BrdU-positive cells in WT muscle were all interstitial. We next counted resident macrophages in frozen tissue sections, identified by F4/80 immunoreactivity, and determined them to be 65.9 ± 15.5 F4/80-positive cells/mm^2^ in Bla/J vs. 5.5 ± 2.5 in WT (Fig. 2a, c), indicating that, consistent with published observations, uninjured Bla/J muscle contains excess macrophages [17]. Finally, we examined uninjured Bla/J muscle for M1 (*Mcp1*, *Ifng*, *Il1b*, *Tnfα*) and M2 (*Ym1*, *Il10*, *Arg1*, *Fizz1*, *IL-4*, and *CCR2)*-specific markers. We observed a significant increase in the expression level of MCP1 and IL-1β genes (*P <* 0.05) in Bla/J muscle, suggesting increased accumulation of M1 macrophages (Fig. 2d). We further observed that whereas the expression level of the *Ym1* gene, a marker of M2a macrophages was significantly increased in Bla/J muscle, the expression level of the *IL-10* gene, a marker of M2b/c macrophages was not significantly increased above age-matched WT muscle (Fig. 2e). These data suggest that both M1 and M2a macrophages constitute the major infiltrating macrophage phenotypes in 4–7-month-old dysferlin-deficient muscle.

**Fig. 3:**
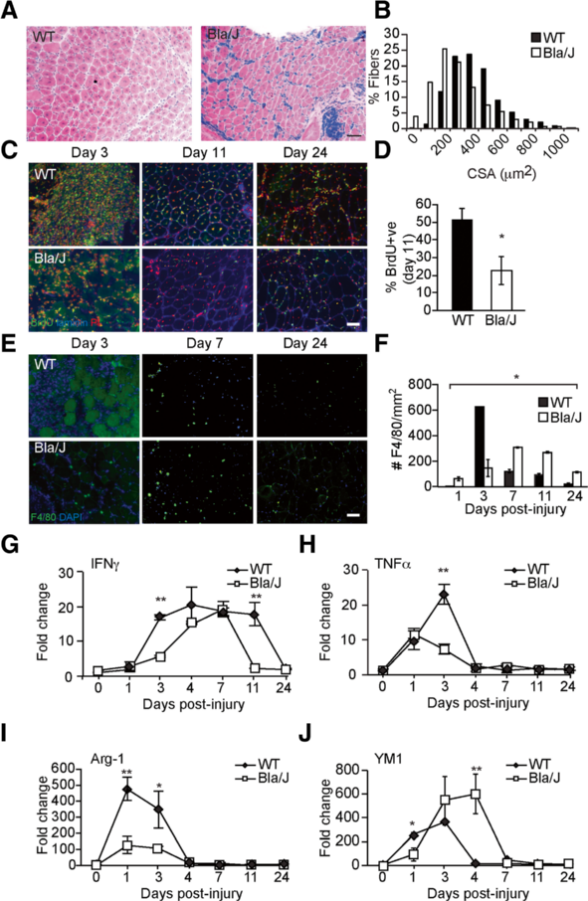
Response to injury is attenuated in Bla/J muscle compared with WT. **a** H&E staining of WT (*left*) and Bla/J (*right*) muscle on day 7 after injury. **b** Fiber cross-sectional areas (CSA) from WT (*black*) and Bla/J (*white*) on day 7 after injury. Fiber diameter distribution of Bla/J muscle is shifted to the left. Residual tattoo ink (*blue staining*) can still be seen in the injured Bla/J muscle. **c** Immunofluorescence staining for BrdU (*green*) and laminin (*blue*) on indicated days following notexin injury in WT or Bla/J TA muscle. Nuclei were detected with propidium iodide (PI, *red*). **d** Quantitation of BrdU-positive nuclei on day 11 after injury shown as percent of BrdU-positive cells over total myonuclei. **e** Immunofluorescence staining for F4/80 (*green*) on indicated days following notexin injury in WT or Bla/J TA muscle. Nuclei were detected with DAPI (*blue*). **f** Number of F4/80-positive macrophages per mm^2^ on indicated days after injury. *n* = 3. Expression of M1-specific markers *IFNγ* (**g**) and *TNFα* (**h**) and M2-specific markers *Arg-1* (**i**) and *YM1* (**j**) using qRT-PCR on indicated days following injury. Data are shown as means ± SEM. *P < 0.05. Scale bar, 50 μm

Enhanced NFκB signaling and inflammation has been shown to inhibit muscle growth and differentiation [26, 27], suggesting that chronic inflammatory processes in dysferlin-deficient muscle impede successful regeneration. To determine how the presence of chronic inflammatory macrophages affects the response to injury, we used the snake venom notexin, a potent and widely used myotoxin [28], to induce muscle injury in 4–7-month-old Bla/J and WT mice. Injection of notexin resulted in a robust regenerative response to injury in WT mice, followed by appearance of centrally nucleated regenerated fibers by day 7 post-injury. Although centrally nucleated regenerated fibers were also apparent in Bla/J muscle, they were of smaller cross-sectional area (CSA) than WT (Fig. 3a, b), with the fiber diameter distribution shifted to the left, indicating a delay in regeneration. Cellular proliferation in response to injury was evaluated using BrdU incorporation, administered in drinking water for 1 week following injury. Analysis of BrdU incorporation in frozen sections revealed BrdU-positive nuclei, which could be detected by day 3, with peak incorporation observed by day 11. At this time point, 51 ± 6% of nuclei were BrdU-positive in WT muscle, whereas only 23 ± 8% of nuclei were BrdU-positive in dysferlin-deficient muscle (Fig. 3c, d). Moreover, assessment of F4/80-positive inflammatory macrophages in the frozen muscle sections showed a robust regenerative response in WT muscle with a peak on day 3 of, on average, 645.8 ± 10.4 cells/mm^2^, followed by a rapid reduction in the infiltrate to an average of 20.8 ± 6.9 cells/mm^2^ by day 24 (Fig. 3e, f). The regenerative response in dysferlin-deficient muscle was attenuated and prolonged, reaching a peak on day 7 with only 319.4 ± 20.8 cells/mm^2^. By day 24, the F4/80-positive cell count remained elevated in Bla/J muscle with 118.0 ± 24.3 cells/mm^2^ remaining, indicating inadequate resolution of the injury response (Fig. 3e, f). The macrophage response to injury was further studied by examining gene expression levels of markers of M1 and M2a macrophages by qRT-PCR throughout the course of recovery. This profiling study revealed lower gene expression of M1 markers (*IFNγ*, *TNFα* and *Arg-1*) in Bla/J than in WT muscle on days 0–3 (Fig. 3g–i). In contrast, gene expression of the M2a macrophage marker, *Ym1*, was greater in Bla/J than in WT muscle (Fig. 3j). These data suggest an attenuated M1, but not M2a, recruitment in response to injury in Bla/J muscle.

### *In vitro* model of myoblast and macrophage interactions

Having shown that macrophages have different effects on WT and dysferlin-deficient muscle, we sought to directly test their cellular interactions. We recently characterized H-2K immortalized myoblasts from dysferlin-deficient (A/J) mice (A/J myoblasts) and demonstrated that intrinsically upregulated NFκB pathway signaling inhibits their differentiation efficiency compared with immortalized H-2K muscle cells derived from dysferlin-sufficient (WT) littermates (WT-myoblasts) [21]. To establish the cellular and molecular effects that macrophage-derived factors exert on muscle cells, we developed an *in vitro* co-culture model of macrophages and myoblasts.

**Fig. 4:**
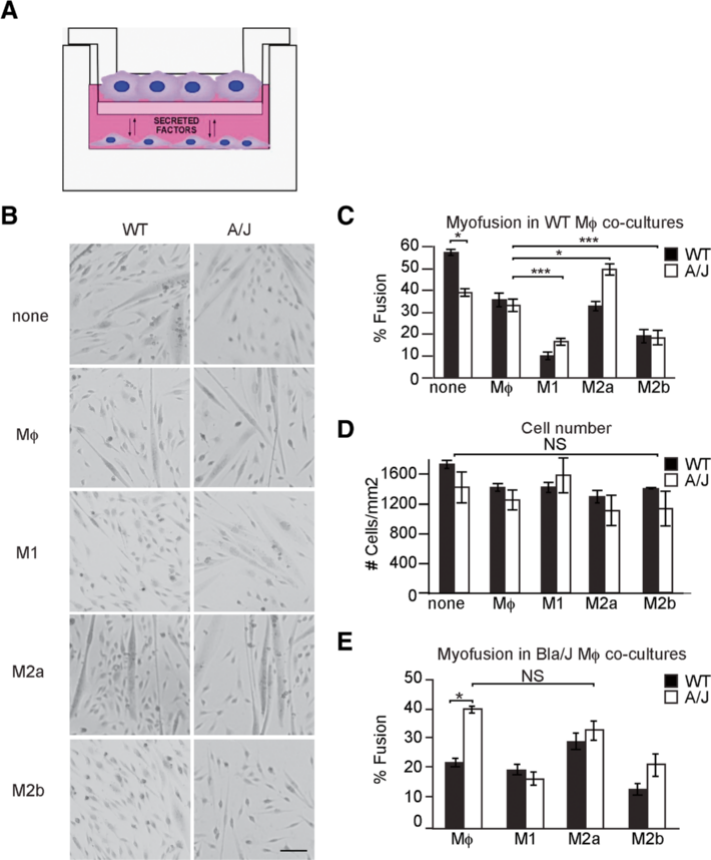
Co-culture of A/J and WT myoblasts with M1 and M2a macrophages. **a** Diagram of myoblast-macrophage co-cultures. H-2K cells are plated in a 6-well dish and macrophages are plated in the top Transwell^TM^ chamber. Soluble factors secreted by both macrophages and myoblasts can be exchanged in the culture medium. **b** Desmin immunostaining of dysferlin-deficient (A/J, *left column*) and dysferlin-sufficient (WT, *right column*) myoblasts after 3 days of co-culture with differentially polarized macrophages: LPS treated (M1), IL-4 treated (M2a), LPS+OVA-IC treated (M2b), or untreated (Mϕ). Scale, 50 μm. **c** % Fusion index in 3-day-old co-cultures of WT and A/J myoblasts with WT mice-derived macrophages obtained by dividing the number of nuclei in myotubes by total myonuclei. **d** Total number of cells in 3-day-old co-cultures with WT mice-derived macrophages. **e** % Fusion index in 3-day-old co-cultures of WT and A/J myoblasts with Bla/J mice-derived macrophages. *n* = 3 independent experiments. Data are shown as means ± SEM; ANOVA, *P < 0.05; ***P < 0.001

The cellular effects of macrophages on dystrophic muscle cells have not been characterized previously. Initial experiments with co-plating showed that direct contact with macrophages diminished myoblast viability. To look for more subtle effects on muscle, we developed a Transwell co-culture in which only secreted factors are exchanged. WT or A/J myoblasts were plated onto Matrigel^™^-coated dishes and, 1 day later, differentiated macrophages were added onto the Transwell inserts (Fig. 4a).

Bone marrow-derived macrophages (Mϕ) were differentiated to induce three macrophage phenotypes: classically activated (M1), alternatively activated (M2a), and regulatory macrophages (M2b), as previously described (see the “Methods” section; [19]). Activation of the M1 phenotype was confirmed by increased gene expression of *IL-12* (Fig. 1a), *TNFα* (Fig. 1b), and *iNOS* (Fig. 1c) and secretion of IL12p40 ([19]; Fig. 1d), 24 h after stimulation. Activation of the M2a phenotype was confirmed by increased expression of *Fizz-*1 ([18]; Fig. 1e) and *IL-4* (Fig. 1f).

### WT M1 macrophages inhibit, while M2a macrophages potentiate myogenic differentiation of A/J cells

Following 3 days of co-culture, muscle differentiation was assessed by immunostaining for the muscle-specific marker, desmin (Fig. 4b). WT myoblasts cultured in the absence of macrophages differentiated well following the 3-day differentiation period with 57.5 ± 1.4 % nuclei being in myotubes (none, Fig. 4c). Differentiation was lower in A/J myoblasts cultured alone (39.1 ± 1.9 %, *P* < 0.05), as had been previously reported [21]. Co-culture of both WT and A/J myoblasts with M1 or M2b macrophages significantly reduced myofusion (Fig. 4c). However, the reduction to myofusion observed in WT myoblast-M1 macrophage co-cultures (83 % reduction, from 57.5 ± 1.4 to 9.9 ± 1.8 %) was greater than that observed in A/J myoblast-M1 macrophage co-cultures (58 % reduction, from 39.1 ± 1.9 to 16.5 ± 1.6 %) (Fig. 4c). Total cell numbers of WT and A/J cells were unaffected by co-culture (Fig. 4d).

Co-culture with M2a macrophages also had different effects on WT and A/J cells, whereas co-culture with M2a macrophages significantly increased fusion of A/J myoblasts, without change in total cell numbers (39.0 ± 1.9 vs. 49.7 ± 2.5 %, *P* < 0.05) (Fig. 4c, d), such an increase was not observed in WT myoblast-M2a macrophage co-cultures (57.5 ± 1.4 vs. 32.7 ± 2.2 %, *P* < 0.05). Since none of these effects resulted from changes to cell numbers (Fig. 4d), these data suggest that differentiation of A/J myoblasts is resistant to the anti-myogenic effects of M1 macrophages, but may be potentiated by the pro-myogenic effects of M2a macrophages.

### Dysferlin-deficient M2a macrophages do not potentiate myogenic differentiation of A/J myoblasts

Dysferlin-deficient murine macrophages were previously reported to be more phagocytic than WT [29]. We used the co-culture model to further study the effects of macrophages derived from dysferlin-deficient mice (Bla/J) on muscle differentiation, compared with macrophages derived from C57Bl/6J mice (WT). WT myoblasts co-cultured with unpolarized Bla/J Mϕ macrophages showed approximately 37 % less myotube fusion (22.0 ± 1.3 %) (Fig. 4e) than those co-cultured with WT Mϕ macrophages (35.6 ± 3.1 %) (Fig. 4c). Interestingly, such inhibition of myotube fusion was not observed when A/J myoblasts were co-cultured with Bla/J Mϕ macrophages. Furthermore, in contrast to the beneficial effects on A/J myoblasts of WT M2a macrophage co-culture, their fusion was not improved by co-culture with Bla/J M2a macrophages (Fig. 4e). Together, these data suggest that M2a macrophages from Bla/J mice show reduced pro-myogenic activity.

### Dysferlin-deficient macrophages show enhanced IL-10 and IL-12 expression

Our analyses suggested that the observed anti-myogenic effects of M1 macrophages and pro-myogenic effects of M2a macrophages are mediated by soluble factors secreted from each respective phenotype. Thus, we sought to identify the factors mediating the effects on muscle differentiation in the co-cultures.

**Fig. 5:**
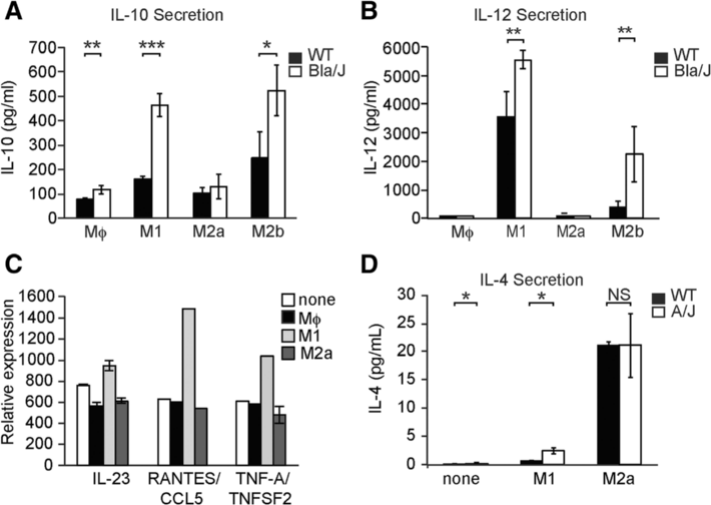
Cytokines secreted in myoblast-macrophage co-cultures. ELISA analysis of 3-day old WT co-culture supernatants showing expression of IL-10 (**a**) and IL-12 (**b**) in differentially polarized macrophages from WT (*black bars)* or Bla/J (*white bars*) mice. **c** Cytokine arrays performed on supernatants of 3-day-old WT myoblasts co-cultured without macrophages (*white*) or with WT mice-derived Mϕ (*black*), M1 (*light grey*), or M2a (*dark grey*) macrophages showing expression of M1-specific cytokines. **d** MSD IL-4 assay performed on supernatants of 1-day-old WT (*white*) or A/J (*black*) myoblasts that were either cultured alone (none) or co-cultured with WT mice-derived M1 or M2a macrophages. Data are shown as means ± SEM. *n* = 3; *P < 0.05; **P < 0.01; ***P < 0.001

The marked differences of WT and Bla/J macrophages on myoblast differentiation might be due to intrinsic differences in cytokine secretion. We focused on comparing two key factors in the co-cultures: IL-12 secreted in response to LPS (M1) stimulation and IL-10 secreted in response to LPS+OVA-IC (M2b). ELISA analysis performed on supernatants from 3-day-old co-cultures showed that Bla/J macrophages released more IL-10 than WT in response to LPS (*P* < 0.001) and LPS+OVA-IC (*P* < 0.05) stimulation and more IL-12 in response to LPS (*P* < 0.01) and LPS+OVA-IC (*P* < 0.01) stimulation (Fig. 5a, b). These data demonstrate that Bla/J macrophages release more pro-inflammatory cytokines.

Finally, we used cytokine arrays to analyze the soluble factors released into the culture medium after 3 days of WT myoblast co-culture. Analysis of conditioned medium of WT cells co-cultured with M1 macrophages revealed expression of pro-inflammatory cytokines IL-23, RANTES, and TNFα (Fig. 5c). In contrast, co-culture with M2a macrophages resulted in secretion of the Th2-associated cytokine, IL-4. We further determined the concentration of IL-4 in the M2a co-cultures to be unchanged between WT and A/J myoblasts (10.6 ± 0.3 vs. 10.6 ± 2.8 pg/ml, respectively) (Fig. 5d).

### M1 and M2a macrophages exert different molecular effects on WT and dysferlin-deficient myoblasts

**Fig. 6:**
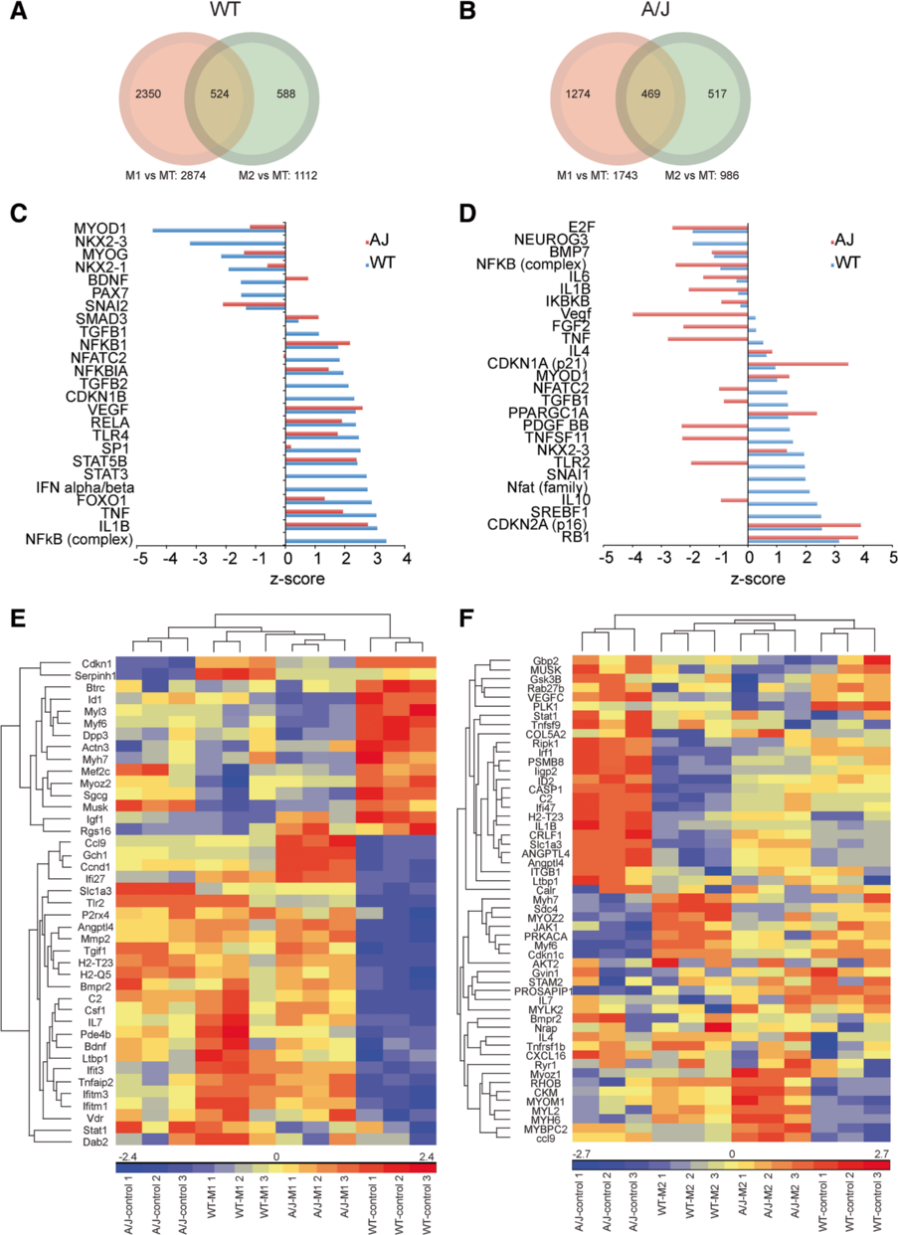
Illumina Gene array analysis of myoblasts co-cultured with macrophages. Venn diagrams showing gene subsets in WT (**a**) or A/J (**b**) myoblasts that were differentially regulated by co-culture with M1 only (*green*), by M2a only (*red*), or in both co-cultures (*overlap*). ANOVA, *P* < 0.01. Transcriptional networks activated by co-culture with M1 (**c**) or M2a (**d**) in A/J (*red*) and WT (*blue*) myoblasts sorted by z-score. z-score >1.5 or <1.5 indicates activated and inhibited networks, respectively. **e**, **f** Partek clustering analysis showing genes expressed in WT control myoblasts, A/J control myoblasts and WT or A/J myoblasts co-cultured with M1 (**e**), or M2a (**f**). *Blue* indicates downregulated; *red* indicates upregulated

The co-culture experiments suggested that M1 and M2a-polarized macrophages can greatly impact the course of myoblast differentiation, and this activity is dependent on functional dysferlin both in macrophages and in myoblasts. To gain insight into the molecular events orchestrating these effects, we performed Illumina transcriptome analysis on WT and A/J myoblasts co-cultured for 3 days with M1 or M2a macrophages derived from BALB/c mice (Fig. 6). To identify transcripts in differentiated muscle cultures regulated specifically by co-culture with either M1 or M2a macrophages, transcriptome data were normalized to myoblasts cultured alone (untreated). We identified 2874 transcripts that were differentially regulated in WT myoblasts co-cultured with M1 macrophages and 1112 transcripts that were differentially regulated in WT myoblasts co-cultured with M2a macrophages (*P* < 0.01) (Fig. 6a). Venn diagrams showed that, of these, 2350 transcripts were regulated specifically by WT myoblast-M1 macrophage co-culture, 588 were regulated specifically by WT myoblast-M2a macrophage co-culture, and 524 transcripts were in common within WT myoblast-M1 macrophage and WT myoblast-M2a macrophage co-cultures (Fig. 6a). Interestingly, there were fewer regulated transcripts in A/J myoblasts co-cultured with macrophages: 1743 transcripts were differentially regulated in A/J myoblast-M1 macrophage co-cultures and 986 transcripts were differentially regulated in A/J myoblast-M2a macrophage co-cultures (Fig. 6b). Venn analysis showed that, of these, 1274 transcripts were regulated specifically by A/J myoblast-M1 macrophage co-culture, 517 were regulated specifically by A/J myoblast-M2a macrophage co-culture, and 469 were regulated by both A/J myoblast-M1 macrophage and A/J myoblast-M2a macrophage co-cultures (*P* < 0.01). Thus, co-culture with M1 macrophages had less of an impact on the transcriptome of A/J than WT myoblasts (Fig. 6b).

Ingenuity Pathway Analysis led to the identification of the major transcriptional networks that were differentially regulated in WT myoblasts co-cultured with M1 macrophages. The major upregulated networks included the pro-inflammatory NFκB, IL-1β, TNFα, and TGFβ1 networks (Fig. 6c), while the major downregulated networks were muscle-specific networks including MyoD1, myogenin, and Pax7 (Fig. 6c). We had previously shown that gene expression of *Il1β* is increased in dysferlin-deficient A/J myoblasts [21]. Consistent with the prior report, *Il1β* was upregulated by 3.0-fold (*P* < 0.001) in untreated A/J compared with untreated WT myoblasts. Moreover, the IL-1β, NFκB, TNFα, and TGFβ1 networks were less affected in M1-co-cultured A/J myoblasts than in WT, consistent with those networks being intrinsically upregulated in untreated A/J myoblasts (Fig. 6c). Partek clustering analysis also showed that untreated A/J myoblasts clustered with, i.e., were more similar to, WT myoblast-M1 macrophage co-cultures rather than untreated WT myoblasts (Fig. 6e). Co-culture of WT myoblasts with M1 macrophages led to significant upregulated expression of pro-inflammatory genes including *C2*, *Csf1*, *Ifitm1*, *Tnfaip2*, and *Tlr2* (Fig. 6e, Table 2). Conversely, co-culture of WT myoblasts with M1 macrophages led to decreased expression of muscle differentiation-specific genes, including *Actn3*, *Igf1*, *Myoz2*, and *Myh7* (Fig. 6e, Table 2). In contrast to WT, pro-inflammatory genes were upregulated, and muscle differentiation genes were downregulated, in untreated A/J myoblasts, compared with untreated WT myoblasts, and incubation with M1 macrophages did not further modify their expression (Fig. 6e, Table 2). These data suggest that intrinsically upregulated pro-inflammatory networks in A/J muscle cultures are resistant to further activation by co-culture with M1 macrophages.

**Table 2:** Transcripts modulated in A/J vs. WT myoblasts co-cultured with M1 macrophages

Gene symbol	Description	WT-M1 vs. WT	AJ vs. WT	AJ-M1 vs. AJ
*Ifi27*	Interferon, alpha-inducible protein 27	16.32	2.41	17.75
*Ifit3*	Interferon-induced protein with tetratricopeptide repeats 3	10.49	1.75	3.44
*C2*	Complement component 2	2.88	2.63	n/a
*Ifitm1*	Interferon-induced transmembrane protein 1	2.86	1.76	n/a
*MMP2*	Matrix metallopeptidase 2	2.77	2.54	n/a
*IL7*	Interleukin 7	2.62	1.69	n/a
*H2-T23*	Histocompatibility 2, T region locus 23	2.56	4.41	n/a
*Ifitm3*	Interferon-induced transmembrane protein 3	2.50	1.65	n/a
*Vdr*	Vitamin D (1,25-dihydroxyvitamine D3) receptor	2.37	2.18	n/a
*Tnfaip2*	Tumor necrosis factor, alpha-induced protein 2	2.28	1.57	n/a
*Csf1*	Colony stimulating factor 1	2.25	1.81	n/a
*CCL9*	Chemokine (C-C motif) ligand 9	2.23	1.99	2.79
*Ltbp1*	Latent transforming growth factor beta binding protein 1	2.20	1.53	n/a
*Pde4b*	Phosphodiesterase 4B, cAMP-specific	2.14	1.61	1.73
*H2-Q5*	Histocompatibility 2, Q region locus 5	2.00	2.63	n/a
*Slc1a3*	Solute carrier family 1 (glial high affinity glutamate transporter), member 3	1.99	5.59	n/a
*Angptl4*	Angiopoietin-like 4	1.98	1.93	n/a
*Gch1*	GTP cyclohydrolase 1	1.94	2.48	3.14
*Bdnf*	Brain-derived neurotrophic factor	1.93	1.54	n/a
*P2rx4*	Purinergic receptor P2X, ligand-gated ion channel 4	1.86	1.82	n/a
*Tgif1*	TGFB-induced factor homeobox 1	1.82	2.38	n/a
*Serpinh1*	Serpin peptidase inhibitor, clade H (heat shock protein 47)	1.76	1.53	1.64
*Stat1*	Signal transducer and activator of transcription 1	1.67	1.77	−1.72
*Ccnd1*	Cyclin D1	1.64	1.57	1.66
*Dab2*	Disabled 2, mitogen-responsive phosphoprotein	1.62	1.79	n/a
*Nfkbie*	Nuclear factor of kappa light polypeptide gene enhancer in B-cells inhibitor, epsilon	1.60	n/a	1.54
*Tlr2*	Toll-like receptor 2	1.58	1.75	n/a
*Bmpr2*	Bone morphogenetic protein receptor, type II	1.50	1.58	n/a
*Actn3*	Actinin, alpha 3	−1.50	−1.50	n/a
*Tlr4*	Toll-like receptor 4	−1.53	n/a	−1.56
*Rgs16*	Regulator of G-protein signaling 16	−1.54	−1.64	n/a
*Dpp3*	Dipeptidyl-peptidase 3	−1.56	−1.63	n/a
*Myh7*	Myosin, heavy chain 7 (type I)	−1.65	−1.87	n/a
*Id1*	Inhibitor of DNA binding 1	−1.72	−1.53	−1.59
*IL1b*	Interleukin 1 beta	−1.73	3.05	−2.89
*Cdkn1c*	Cyclin-dependent kinase inhibitor 1C (p57, kip2)	−1.75	−18.10	2.61
*Mef2c*	Myocyte enhancer factor 2C	−1.80	−1.86	−2.27
*Btrc*	Beta-transducin repeat containing E3 ubiquitin protein ligase	−1.89	−1.50	n/a
*Igf1*	Insulin-like growth factor 1	−1.91	−1.71	n/a
*Tgfb1*	Transforming growth factor, beta 1	−1.96	1.58	−2.56
*Tgfb2*	Transforming growth factor, beta 1	−2.00	n/a	−1.84
*Musk*	Muscle, skeletal, receptor tyrosine kinase	−2.03	−4.73	−1.90
*Myoz2*	Myozenin 2	−2.88	−1.68	n/a
*Sgcg*	Sarcoglycan, gamma (35kDa dystrophin-associated glycoprotein)	−3.01	−1.83	−1.72
*Myl3*	Myosin, light chain 3, skeletal slow	−3.07	−2.42	−1.53
*Myf6*	Myogenic factor 6	−4.13	−3.53	−1.94
*Myh1*	Myosin, heavy chain 1 (type IIx/d)	−5.89	1.91	n/a

Fold-change values of Illumina Gene array transcript subset which showed significant modulation by WT-M1 co-culture vs. WT alone compared to their fold-change values in A/J vs. WT and A/J-M1 co-culture vs. A/J alone. Only transcripts with fold-change values >1.5 and *P* values <0.01 were considered for analysis. ANOVA was applied to verify significance

Co-culture of WT myoblasts with M2a macrophages activated muscle-specific, and inhibited inflammatory, networks (Fig. 6d). Interestingly, muscle-specific networks including *Rb1* and *Cdkn2a* were activated to a greater extent in A/J than WT myoblasts when co-cultured with M2a macrophages. Moreover, several pro-inflammatory networks, including NFκB, IL-6, and IL-1β, that were initially higher in untreated A/J than WT myoblasts, were decreased to a greater extent than WT when co-cultured with M2a macrophages (Fig. 6d). Further, clustering analysis showed that co-culture of A/J cells with M2a macrophages “ameliorated” their characteristic pro-inflammatory phenotype by down-regulating expression of pro-inflammatory genes, including *Tnfsf9*, *Irf1*, *C2*, and *Il1b*, and up-regulating the expression of muscle-specific genes, including *Myoz1*, *Myl2*, *Myh6*, *Myf6*, and *Musk*. Thus A/J myoblast-M2a macrophage co-cultures clustered closely with, i.e., were more similar to, both untreated WT myoblasts and WT myoblast-M2a macrophage co-cultures (Fig. 6f, Table 3). These analyses support the idea that M2a macrophages (or factors released from them) may improve the differentiation of dysferlin-deficient muscle cells by inhibiting the intrinsic pro-inflammatory environment that is characteristic of dysferlin-deficient muscle.

**Table 3:** Transcripts modulated in A/J vs. WT myoblasts co-cultured with M2a macrophages

Gene symbol	Description	WT-M2 vs. WT	AJ vs. WT	AJ-M2 vs. AJ
*Myl2*	Myosin, light polypeptide 2, regulatory, slow	2.31	n/a	3.07
*Sdc4*	Syndecan 4	2.04	−2.16	n/a
*Nrap*	Nebulin-related anchoring protein	1.98	n/a	2.29
*Calr*	Calreticulin	1.88	n/a	−1.87
*Mylk2*	Myosin light chain kinase 2	1.88	n/a	n/a
*Ltbp1*	Latent transforming growth factor beta binding protein 1	1.85	1.54	n/a
*Ckm*	Creatine kinase, muscle	1.83	n/a	1.78
*Mybpc2*	Myosin binding protein C, fast type	1.64	2.47	1.94
*Rhob*	Ras homolog family member B	1.62	n/a	1.58
*Myh7*	Myosin, heavy chain 7 (type I)	1.61	−1.87	n/a
*Il4*	Interleukin 4	1.59	n/a	n/a
*Ryr1*	Ryanodine receptor 1	1.57	n/a	n/a
*Myoz2*	Myozenin 2	1.56	−1.69	1.81
*Myh6*	Myosin, heavy chain 7 (cardiac)	1.52	n/a	2.00
*Myom1*	Myomesin 1	1.52	n/a	1.54
*Id2*	Inhibitor of DNA binding 2	−1.56	1.77	−2.20
*Il7*	Interleukin 7	−1.57	1.70	n/a
*Rab27b*	RAB27B, member RAS oncogene family	−1.61	n/a	−1.67
*Iigp2*	Interferon inducible GTPase 2	−1.79	2.06	−2.83
*Psmb8*	Proteosome subunit, beta type 8	−1.84	1.78	−1.86
*Ifi47*	Interferon gamma inducible protein 47	−1.90	2.18	−2.00
*C2*	Complement 2	−1.95	2.64	−2.40
*Casp1*	Caspase 1	−2.18	1.98	−2.22
*Plk1*	Polo-like kinase 1	−2.29	−2.76	−1.68
*Irf1*	Interferon regulatory factor 1	−2.91	1.88	−2.43
*Gbp2*	Guanylate binding protein 2, interferon-inducible	−3.37	1.60	−2.33
*Akt2*	v-akt murine thymoma viral oncogene 2	n/a	n/a	1.51
*Gsk3B*	Glycogen synthase kinase 3	n/a	n/a	−1.75
*Itgb1*	Integrin, beta 1 (fibronectin receptor beta)	n/a	n/a	−2.37
*Jak1*	Janus kinase 1	n/a	n/a	−1.87
*Myoz1*	Myozenin 1	n/a	n/a	1.70
*Ripk1*	Receptor (TNFRSF)-interacting serine-threonine kinase 1	n/a	n/a	−1.51
*Vegfc*	Vascular endothelial growth factor C	n/a	n/a	−1.50
*Slc1a3*	Solute carrier family 1 (glial high affinity glutamate transporter), member 3	n/a	5.59	−1.98
*H2-T23*	Histocompatibility 2, T region locus 23	n/a	4.41	−1.66
*Il1b*	Interleukin 1 beta	n/a	3.05	−2.47
*Cxcl16*	Chemokine (C-X-C motif) ligand 16	n/a	2.92	−1.74
*Col5a2*	Collagen, type V, alpha 2	n/a	2.66	−1.72
*Crlf1*	Cytokine receptor-like factor 1	n/a	2.64	−1.69
*Ccl9*	Chemokine (C-C motif) ligand 9	n/a	2.00	1.50
*Angptl4*	Angiopoietin-like 4	n/a	1.93	−1.52
*Stat1*	Signal transducer and activator of transcription 1	n/a	1.77	−1.61
*Tnfrsf1b*	Tumor necrosis factor receptor superfamily, member 1b	n/a	1.63	−1.51
*Bmpr2*	Bone morphogenetic protein receptor 2	n/a	1.58	−1.61
*Stam2*	Signal transducing adaptor molecule (SH3 domain and ITAM motif) 2	n/a	1.57	−1.62
*Gvin1*	GTPase, very large interferon inducible 1	n/a	1.53	−1.75
*Tnfsf9*	Tumor necrosis factor (ligand) superfamily, member 9	n/a	1.51	−1.57
*Prkaca*	Protein kinase, cAMP-dependent, catalytic, alpha	n/a	−1.58	1.51
*Prosapip1*	ProSAPiP1 protein	n/a	−1.90	1.67
*Myf6*	Myogenic factor 6	n/a	−3.54	2.62
*Musk*	Muscle, skeletal, receptor tyrosine kinase	n/a	−4.73	−1.53
*Cdkn1c*	Cyclin-dependent kinase inhibitor 1C (p57, Kip2)	n/a	−18.11	5.52

Fold-change values of Illumina Gene array transcript subset which showed significant modulation by WT-M2a co-culture vs. WT alone compared to their fold-change values in A/J vs. WT and A/J-M2a co-culture vs. A/J alone. Only transcripts with fold-change values >1.5 and *P* values <0.01 were considered for analysis. ANOVA was applied to verify significance

**Fig. 7:**
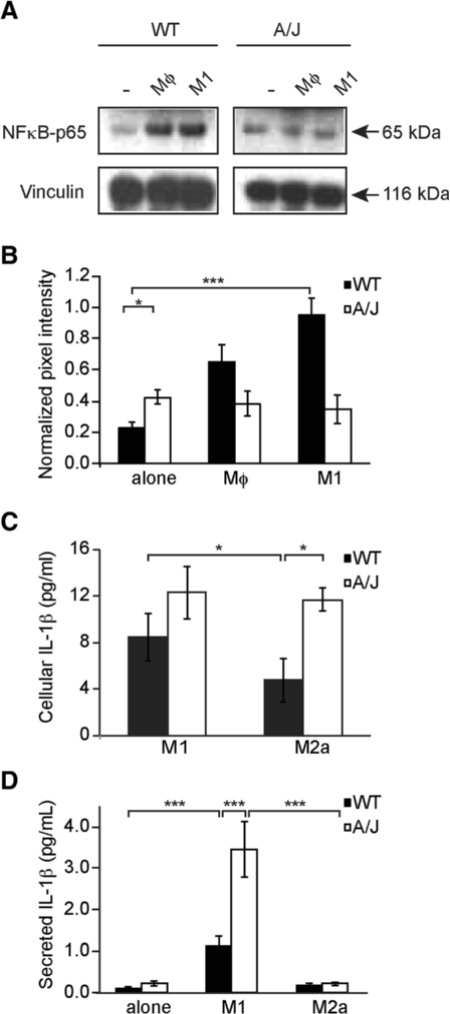
NFκB and IL-1β in myoblast-macrophage co-cultures. **a** Representative Western blot showing phosphorylation of the NFκB p65 subunit in WT or A/J myoblasts either cultured alone (-), or co-cultured for 3 days with Mϕ, or M1 macrophages. **b** Quantitation of phospho-p65 pixel intensity in **a**, normalized to vinculin pixel intensity. *n* = 3 independent experiments. **c** IL-1β ELISA analysis of whole cell lysates prepared from WT (*black*) or A/J myotubes (*white*) co-cultured for 24 h with M1 or M2a macrophages. **d** MSD IL-1β assay performed on supernatants from WT (*black*) or A/J myotubes (*white*) co-cultured for 24 h alone, with M1 or M2a macrophages. Two-way ANOVA with Tukey post hoc test were used to calculate *P* values. *n* = 3 cultures per data point. Data are shown as means ± SEM. *P < 0.05; ***P < 0.001

The data from the desmin staining and transcriptome analyses suggested a blunted response in A/J myoblasts to co-culture with M1 macrophages, suggesting that A/J myoblasts may be less sensitive than WT to the pro-inflammatory effects of M1 macrophages. We tested this idea by examining NFκB pathway activation in lysates of WT and A/J myoblasts co-cultured with M1 macrophages (Fig. 7). In accord with the transcriptome analysis, co-culture of WT myoblasts with M1 macrophages led to robust phosphorylation of the NFκB-p65 subunit (phospho-p65) (Fig. 7a, b), whereas phospho-p65, which was significantly upregulated in untreated A/J cultures, was not further augmented by co-culture with either Mϕ or M1 macrophages (Fig. 7a, b).

The transcriptome analysis showed that IL-1β gene expression is upregulated in A/J myoblasts. Therefore, we tested for expression of IL-1β protein in lysates of WT and dysferlin-deficient co-cultured myotubes using an ELISA assay that detects both the precursor and mature forms of IL-1β. When WT myoblasts were co-cultured with M2a macrophages, they expressed low levels of IL-1β but showed significant upregulation of cellular IL-1β when co-cultured with M1 macrophages. Compared with WT myoblasts, expression of IL-1β in A/J myoblasts was significantly upregulated in M2a macrophage co-cultures and was not further enhanced in M1 macrophage co-cultured myotubes (Fig. 7c). We further tested IL-1β secretion into the co-culture supernatants and found that M1 macrophage co-cultures secreted IL-1β at higher levels compared with untreated or M2a macrophage co-cultures, consistent with previous reports [30]. Furthermore, A/J myoblast-M1 macrophage co-cultures secreted more IL-1β when compared with WT myoblast-M1 macrophage co-cultures (Fig. 7d).

### IL-4 potentiates muscle differentiation in both WT and A/J myoblasts

**Fig. 8:**
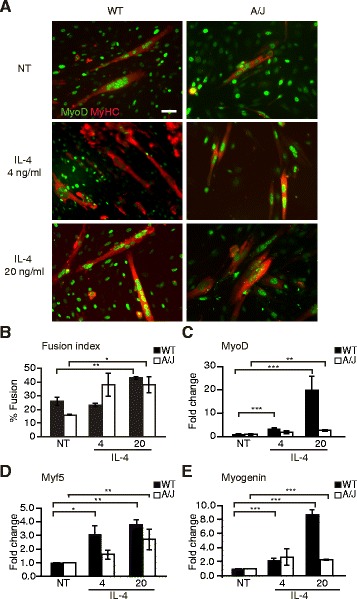
IL-4 potentiates muscle differentiation in WT and A/J myoblasts. **a** Immunofluorescence staining of differentiated WT and A/J cultures treated for 72 h with 4 or 20 ng/ml of IL-4. Control cultures were treated with equal volumes of PBS (NT). Following incubation, cultures were immunostained with anti-MyoD (*green*) and anti-MyHC (*red*). Scale bar, 50 μm. **b** Myofusion index (expressed as % fusion). **c**–**e** Gene expression of *MyoD* (**b**), *Myf5* (**c**), and *myogenin* (**d**) determined by qRT-PCR in WT and A/J cultures treated with indicated concentrations of IL-4. *N* = 4. Data are shown as means ± SEM. ANOVA with Tukey post hoc analysis, *P < 0.05; **P < 0.01; ***P < 0.005

The major M2a macrophage-secreted cytokine identified in our cytokine analyses, IL-4, has previously been implicated in playing an important role in myoblast differentiation, and its loss results in smaller myotubes with fewer myonuclei [31]. However, the effect of IL-4 on myogenic differentiation of dystrophic muscle has not been previously explored. Based on the above studies, we hypothesized that IL-4 might be the soluble factor mediating the beneficial effects of M2a co-culture on A/J myoblasts. To test this possibility, we queried whether IL-4 alone can mirror the beneficial effects of A/J myoblast-M2a macrophage co-culture, by potentiating differentiation. Treatment with 20 ng/ml of IL-4 nearly doubled the percent fusion, compared with untreated, in both WT (43.0 ± 0.8 vs. 26.1 ± 2.9 %, *P* < 0.01) and A/J (38.0 ± 6.0 vs. 15.7 ± 0.1 %, *P* < 0.05) cultures (Fig. 8a, b). Treatment with lower amounts of IL-4 (4 ng/ml) also showed a trend for increase, though not significant. Additionally, treatment with IL-4 potentiated gene expression of *MyoD*, *Myf5*, and *myogenin* in WT muscle cultures (Fig. 8c–e). IL-4 also increased the expression of *MyoD*, *Myf5*, and *myogenin* genes in A/J cultures, but not to the levels observed in WT cultures (Fig. 8c–e). Thus, IL-4 alone is not sufficient to restore the muscle differentiation defect in A/J myoblasts.

### Inhibition of IL-1β ameliorates muscle differentiation in A/J myoblasts

Having shown that IL-4 activity alone is not sufficient to rescue muscle differentiation in A/J muscle cells, we next focused on factors released from M1 macrophages that inhibit muscle differentiation. We initially examined differentiation in cultures treated with the pro-inflammatory cytokines we observed being released by M1 macrophages: IL-10, IL-12, and TNFα, but did not observe any effects on myotube fusion.

**Fig. 9:**
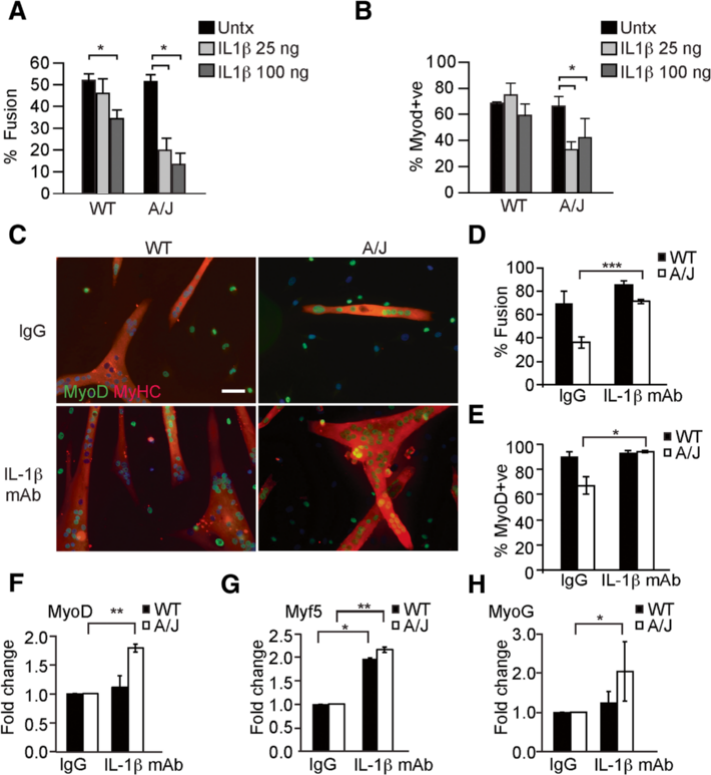
Upregulated IL-1β inhibits muscle differentiation in A/J myoblasts. **a** Quantitation of myotube fusion in WT or A/J myoblasts treated with indicated concentrations of IL-1β for 72 h. Percent myotube fusion was calculated from MyHC-positive immunostaining. **b** Quantitation of MyoD-positive cells are shown as percent of total nuclei in WT or A/J myoblast cultures treated with indicated concentrations of IL-1β for 72 h. **c** Immunofluorescence staining of myoblast cultures after treatment with an IL-1β blocking antibody (IL-1β mAb). WT and A/J cultures were plated at equal density and treated with 4 ng/ml of IL-1β mAb for 72 h. Control cultures were treated with equimolar concentrations of mouse IgG. Fixed cultures were stained with anti-MyoD (green) and anti-MyHC (red) antibodies. **d** Quantitation of myotube fusion in treated cultures. **e** Quantitation of MyoD-positive cells in treated cultures. **f**–**h** Gene expression of *MyoD* (**f**), *Myf5* (**g**), and *myogenin* (**h**) determined by qRT-PCR in cultures treated with indicated concentrations of IL-1β mAb. *n* = 3. Data are shown as means ± SEM. *P < 0.05; **P < 0.01; ***P < 0.005

We were then guided by our transcriptome analysis of co-cultured myoblasts which revealed increased expression of the IL-1β gene in A/J myoblasts (Table 2), as well as our observations of increased cellular and secreted IL-1β protein in A/J myoblasts (Fig. 7c), and qRT-PCR data showing that IL-1β is upregulated in vivo in uninjured Bla/J muscle (Fig. 2). This, together with our previous demonstrations that IL-1β is a major pro-inflammatory factor released from LPS/BzATP-stimulated macrophages [30], and that A/J muscle shows upregulated IL-1β secretion and IL-1β signaling [30, 21] heavily implicated IL-1β in the suppression of muscle differentiation and has not been previously reported. To test this idea *in vitro*, we treated differentiating WT and A/J myoblasts with recombinant IL-1β for 5 days. Treatment with IL-1β reduced the percent fusion and the number of MyoD-positive cells, compared with untreated (Fig. 9a, b). Importantly, the decrease in myotube fusion and MyoD-positive cell number was significantly greater in A/J than WT cultures, indicating that A/J myoblasts are more sensitive than WT to IL-1β-mediated inhibition of muscle differentiation (Fig. 9a, b). These data suggest that IL-1β is the major M1 macrophage-derived factor that inhibits differentiation of A/J myoblasts.

Having identified upregulated IL-1β signaling in A/J myoblasts, and inhibition of myogenesis due to IL-1β secreted from M1 macrophages in Bla/J muscle, we hypothesized that if IL-1β is mediating the defective myogenesis and regeneration in dysferlin-deficiency, then this effect should be blocked by inhibition of IL-1β in muscle cultures. Differentiating WT and A/J myoblast cultures were treated with an anti-IL-1β monoclonal antibody (mAb) or equimolar concentrations of a control anti-mouse IgG. Treatment with the anti-IL-1β mAb significantly increased myotube fusion (Fig. 9c, d), the number of MyoD-positive cells (Fig. 9e), and expression of *MyoD*, *Myf5*, and *myogenin* (Fig. 9f–h) in A/J muscle cultures, compared with control IgG treatment, indicating that suppression of IL-1β signaling arising from M1 macrophages can restore muscle regeneration in dysferlin deficiency.

## Discussion

### Upregulated pro-inflammatory signaling in dysferlin-deficient myoblasts inhibits muscle differentiation and regeneration

Dysferlin-deficient muscle is marked by inflammatory foci, mononuclear infiltrate, and upregulated NFκB pathway signaling [32, 33]. Mounting evidence suggests that the upregulated IL-1β [30] and upregulated NFκB signaling [21] arises not only from immune cells but also from dysferlin-deficient muscle fibers and cells. But whether the inflammation impedes regeneration, and if so, what aspect of it, has been in debate. Here, we present a systematic evaluation of interactions between muscle and macrophage cells to identify key factors mediating the pro- and anti-myogenic response to macrophages in dystrophic muscle. We used the approach of macrophage-myoblast Transwell co-cultures to show that M1-polarized macrophages, acting in part via IL-1β, activate pro-inflammatory networks in WT myoblasts, which greatly diminishes myoblast differentiation (Fig. 4). In contrast, M2a macrophages, acting in part via IL-4, potentiate muscle regeneration, especially in A/J myoblasts. In addition, we show that the intrinsically upregulated pro-inflammatory networks in dysferlin-deficient muscle make it refractory to the myogenic inhibition by M1 macrophages.

There has been some debate about whether loss of dysferlin results in impaired regeneration. Earlier reports suggested that muscle regeneration after crush injury in SJL/J mice was faster than in BALB/c [34]. Examination of notexin-induced injury in C57Bl10-SJL/J mice concluded that neutrophil recruitment was attenuated, but muscle regeneration was unchanged [35]. More recently, experiments using large-strain injury suggested that A/J mice showed no attenuation in either neutrophil or macrophage infiltration [36]. On the other hand, work in our laboratory and others demonstrated that dysferlin-deficient mice lag in muscle regenerative capacity, compared with dysferlin-sufficient [21, 37, 38]. It is possible that differences in strains and types of injury could account for these discrepancies. Very little muscle pathology was observed in both native and C57Bl10-back-crossed SJL/J mice, which we posited could account for the lack of observation of any regeneration defect, and A/J mice lack complement C5 [17]. Thus, to rule out confounding strain-specific effects unrelated to loss of dysferlin, we conducted our experiments in Bla/J mice compared with C57Bl/6J.

Our findings that regeneration after notexin injury in Bla/J mice is attenuated and does not adequately resolve accords with the conclusions of Chiu et al., although the attribution of this outcome to a defect in neutrophil recruitment rather than monocyte recruitment does not marry with our data. We show that M1 macrophages, although upregulated in uninjured dysferlin-deficient muscle, are not recruited as robustly as in WT muscle, with fewer F4/80-positive cells observed by immunofluorescence staining (Fig. 3) throughout the course of recovery. Although we did not test for neutrophil recruitment, our studies suggest that the M1-mediated response to injury is attenuated in Bla/J muscle, which we attribute to chronic M1 macrophage infiltration and upregulation of M1-derived IL-1β and NFκB in the absence of dysferlin.

The attenuated response to M1 macrophage recruitment in Bla/J mice could impede necrotic tissue clearing in dysferlin-deficient muscle and thus be inhibitory to muscle regeneration. There have been substantial reports demonstrating that recruitment of neutrophils and the removal of necrotic myofibers by M1 macrophages is a pre-requisite to successful regeneration [14]. This view is further substantiated by acute injury studies, in which removal of phagocytes by use of liposome-encapsulated clodronate [39], depletion of CD11b promoter-driven diphtheria toxin [13] or by use of a Ly6C and Ly6G-blocking antibody [40], resulted in delayed regeneration, with ensuing increase in necrotic myofibers, interstitial inflammation, and fat infiltration. Whereas phagocytes are necessary for resolution of acute injury, their role is less clear in chronic inflammatory disease; numerous studies have now documented advantages of blocking some aspects of the inflammatory response to improve pathology, by inhibiting TLRs in *mdx* [23] and dysferlin-deficient mice [41], and by depletion of complement C3 in dysferlin-deficient mice [42]. Our studies suggest the specific depletion or blockade of inflammatory mediators, such as IL-1β, as an alternative strategy to take advantage of M1 macrophage-mediated clearance without the subsequent immunopathology associated with their recruitment.

Macrophages from dysferlin-deficient muscle were previously shown to be more phagocytic than WT [29]. We further characterized macrophages from Bla/J mice as secreting excess IL-12 (M1 response) and IL-10 (M2b response), which complements the earlier studies. Furthermore, undifferentiated macrophages from Bla/J mice inhibited differentiation when co-cultured with WT myoblasts. Together, these studies support the view that dysferlin-deficient un-polarized bone marrow-derived macrophages are “primed” towards the M1 phenotype. Such “priming” is likely to be a result of DAMP signals in dysferlin-deficient muscle (reviewed in [43, 8]). DAMP signals are recognized by TLR receptors which activate intracellular signaling pathways via the adaptor molecule, myeloid differentiation primary response gene 88 (MyD88), thus stimulating macrophage activation (reviewed in [43]).

### Effects of differently polarized macrophages on muscle regeneration

Previous characterization of interactions between macrophages and myoblasts demonstrated that direct co-culture with M1-polarized macrophages decreased fusion of WT myoblasts [16]. Our observations using the Transwell co-cultures of WT myoblasts and M1-polarized macrophages show a similar inhibition of differentiation and are in agreement with those made in the earlier study.

Alternatively, activated macrophages have been shown to potentiate muscle growth. ED2+ macrophages selectively increase myoblast proliferation in muscle cultures [44]. A more current classification identifies IL-4-activated M2a macrophages as pro-myogenic, since direct co-culture of myoblasts with M2a macrophages or M2a-conditioned media increased myotube fusion and the number of myogenin-positive cells [16]. In agreement with these findings, our Transwell co-cultures with M2a macrophages facilitated muscle differentiation of dysferlin-deficient myoblasts (Fig. 4), fitting well with our transcriptome analysis, which suggested that the improvement arises from upregulation of pro-myogenic molecular networks in the myoblasts (Fig. 6).

Our particular advance was the identification of IL-4 as the major factor mediating the pro-myogenic activity of M2a macrophages. IL-4 has been documented as a potent promoter of muscle growth. Treatment of muscle cultures with IL-4 *in vitro* leads to myotube hypertrophy, whereas mice lacking IL-4 or its receptor show reduced muscle size [31]. IL-4 has been shown to inhibit secretion of IL-1β, TNF, and IL-6 from monocytes [45], and when secreted following muscle damage, to inhibit differentiation of adipocyte progenitor cells [12]; thus, the beneficial effect of IL-4 on A/J myoblast differentiation may be mediated by its anti-inflammatory and pro-myogenic activities. These studies, together with our current observations, further substantiate the role of IL-4 as an important player mediating muscle growth.

### IL-1β negatively affects muscle regeneration

Co-culture with M1 macrophages had less of an impact on the transcriptome of A/J than WT myoblasts (Fig. 6b), which our data suggest is due to intrinsically up-regulated expression of cytokines, such as IL-1β in the A/J myoblasts. We have previously shown that dysferlin-deficient muscle shows upregulated expression of the IL-1β gene and protein [30, 21] and have now extended these studies to include upregulated IL-1β gene expression in uninjured dysferlin-deficient muscle (Fig. 2), upregulated IL-1β gene expression in A/J myoblasts (Table 2), increased IL-1β precursor and mature forms in lysates (Fig. 7c), and supernatants from A/J myoblasts co-cultured with M1 macrophages (Fig. 7d), compared with WT myoblasts. Importantly, treatment with an IL-1β mAb to neutralize IL-1β improved differentiation of A/J myoblasts (Fig. 9). Together, these studies make a compelling case for IL-1β as a major player mediating the aberrant response to regeneration in dysferlin deficiency. The secreted form of IL-1β is produced from the precursor pro-peptide by cleavage via the NALP-3 complex [30], a process requiring both a DAMP signal such as LPS as well as an additional signal, such as benzylated ATP (BzATP). LPS stimulation of Mϕ macrophages without the BzATP also leads to release of IL-1β [30], which we observed in our co-culture supernatants (Fig. 7d), albeit at low levels, reflecting the short-lived nature of secreted IL-1β. Both the pro-peptide and mature forms of IL-1β are overproduced in dysferlin-deficient muscle [30] where the second signal likely arises from the dystrophic muscle milieu. The upregulated IL-1β gene expression and pro-peptide production could be a consequence of IL-12, which was secreted from M1 macrophages and upregulated in dysferlin-deficient M1 macrophages (Fig. 5). One possibility for the effectiveness of the IL-1β mAb could be the prevention of an autocrine response in which low levels of IL-1β stimulates its own gene expression, as was recently reported in A431 cells [46]. Thus, treatment with the mAb IL-1β might block IL-1β signaling and restore its own gene expression back to baseline. However, how the loss of dysferlin results in upregulated IL-1β and NFκB signaling in myoblasts explanted from the pro-inflammatory environment of dysferlin-deficient muscle remains unknown and will be addressed in future studies.

## Conclusions

We show for the first time that IL-1β is inhibitory towards muscle differentiation, implicating IL-1β as the factor accounting for attenuated muscle regeneration in dysferlin-deficient muscle. We further show that blocking IL-1β leads to a marked improvement of muscle differentiation *in vitro*, suggesting that blockade of IL-1β in vivo may be a therapeutic target for dysferlin deficiency. Several IL-1β blocking monoclonal antibodies are currently being developed for autoimmune disorders including rheumatoid arthritis, cryopyrin-associated periodic syndrome, diabetes, and gout [47–49]. Our data provides the first evidence that inhibiting IL-1β may improve muscle differentiation in dysferlin deficiency and thus presents a novel therapeutic avenue for treating inflammatory myopathies.

## Abbreviations

ANOVA: Analysis of variance
BrdU: 5′bromo-2′deoxyuridine
CCD: Charge-coupled device
Chi3l3: Chitinase 3-like 3
DAMPs: Danger-associated molecular patterns
DAPI: 4′,6-diamidino-2-phenylindole
DMEM: Dulbecco’s Modified Eagle Medium
ECL: Enhanced chemiluminescence
ELISA: Enzyme-linked immunosorbent assay
H&E: Hematoxylin and eosin
IFNγ: Interferon γ
IL: Interleukin
IPA: Ingenuity Pathway Analysis
IVT: *In vitro* transcription
FBS: Fetal bovine serum
LCCM: L929 cell-conditioned media
LGMD2B: Limb-girdle muscular dystrophy 2B
LPS: Lipopolysaccharide
Mrc1: Mannose receptor
NFκB: Nuclear factor kappa-light-chain-enhancer of activated B cells
qRT-PCR: quantitative reverse transcriptase polymerase chain reaction
PBS: Phosphate-buffered saline
PFA: Paraformaldehyde
PI: Propidium iodide
Retnla: Resistin-like α
RMA: Robust multi-array average algorithm
PVDF: Polyvinylidene fluoride
RNA: Ribonucleic acid
SDS-PAGE: Sodium dodecyl sulfate polyacrylamide gel electrophoresis
TBS: Tris-buffered saline
TA: Tibialis anterior
TNF: Tumor necrosis factor
WT: Wild-type
